# Tetra­kis[tris­(2,2′-bi-1*H*-benzimidazole)nickel(II)] bis­(phosphate) sulfate

**DOI:** 10.1107/S1600536808032571

**Published:** 2008-10-15

**Authors:** Chun-Sheng Ling, Lin Yan

**Affiliations:** a Institute of Pharmacy, Henan University, Kaifeng 475004, People’s Republic of China

## Abstract

The title compound, [Ni(C_14_H_10_N_4_)_3_]_4_(PO_4_)_2_(SO_4_), consists of [Ni(C_14_H_10_N_4_)_3_]^2+^ complex cations (.3. symmetry) and disordered anions (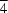 symmetry) with occupancy factors of two-thirds for PO_4_^3−^ and one-third for SO_4_^2−^. The Ni^2+^ centre is chelated by three bidentate 2,2′-bi-1*H*-benzimidazole mol­ecules in a distorted octa­hedral coordination. N—H⋯O hydrogen bonds consolidate the building units into a framework structure.

## Related literature

For the potential applications of metal–organic coordination compounds in gas absorption and separation, catalysis, non-linear optics, luminescence and magnetism, see: Kitagawa & Matsuda (2007 ▶); Maspoch *et al.* (2007 ▶).
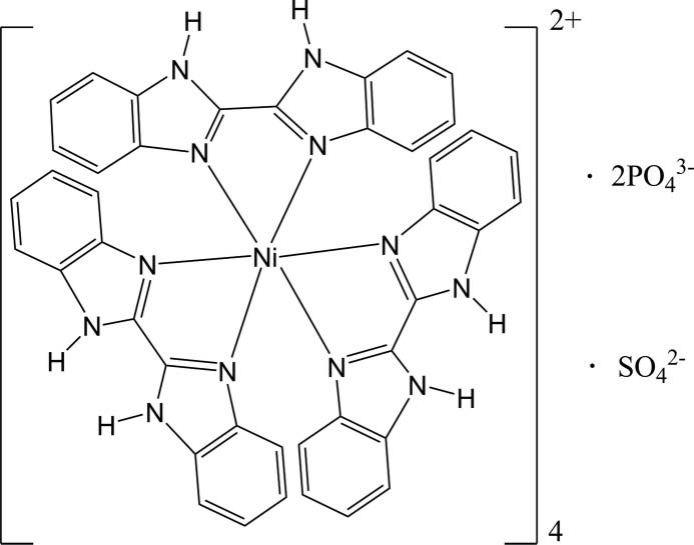


## Experimental

### 

#### Crystal data

[Ni(C_14_H_10_N_4_)_3_]_4_(PO_4_)_2_(SO_4_)
*M* *_r_* = 3331.96
Cubic, 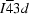

*a* = 24.964 (7) Å
*V* = 15558 (8) Å^3^
*Z* = 4
Mo *K*α radiation
μ = 0.59 mm^−1^
*T* = 296 (2) K
0.32 × 0.27 × 0.23 mm

#### Data collection

Bruker SMART CCD area-detector diffractometer
Absorption correction: multi-scan (*SADABS*; Sheldrick, 2001 ▶) *T*_min_ = 0.834, *T*_max_ = 0.876
20222 measured reflections
2551 independent reflections
1782 reflections with *I* > 2σ(*I*)
*R*_int_ = 0.066

#### Refinement

*R*[*F* ^2^ > 2σ(*F* ^2^)] = 0.038
*wR*(*F* ^2^) = 0.098
*S* = 1.01
2551 reflections
177 parameters
H-atom parameters constrained
Δρ_max_ = 0.61 e Å^−3^
Δρ_min_ = −0.20 e Å^−3^
Absolute structure: Flack (1983 ▶), 1182 Friedel pairs
Flack parameter: −0.02 (2)

### 

Data collection: *SMART* (Bruker, 2001 ▶); cell refinement: *SAINT-Plus* (Bruker, 2001 ▶); data reduction: *SAINT-Plus*; program(s) used to solve structure: *SHELXS97* (Sheldrick, 2008 ▶); program(s) used to refine structure: *SHELXL97* (Sheldrick, 2008 ▶); molecular graphics: *SHELXTL* (Sheldrick, 2008 ▶); software used to prepare material for publication: *SHELXTL*.

## Supplementary Material

Crystal structure: contains datablocks global, I. DOI: 10.1107/S1600536808032571/at2642sup1.cif

Structure factors: contains datablocks I. DOI: 10.1107/S1600536808032571/at2642Isup2.hkl

Additional supplementary materials:  crystallographic information; 3D view; checkCIF report

## Figures and Tables

**Table 1:** Hydrogen-bond geometry (Å, °)

*D*—H⋯*A*	*D*—H	H⋯*A*	*D*⋯*A*	*D*—H⋯*A*
N4—H4*B*⋯O1^i^	0.86	1.96	2.766 (4)	156
N2—H2*B*⋯O1^ii^	0.86	1.82	2.675 (4)	170

Symmetry codes: (i) 
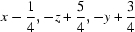
; (ii) 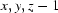.

**Fig. 1.**
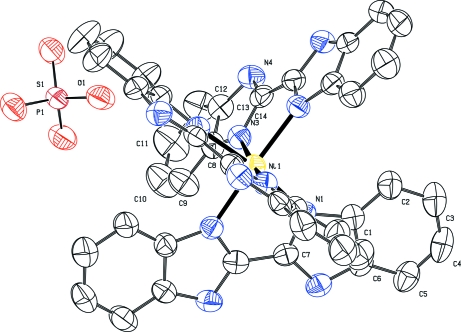
The molecular structure of (I), showing the atom-labelling scheme. Displacement ellipsoids are drawn at the 50% probability level. H atoms are omitted for clarity.

**Fig. 2.**
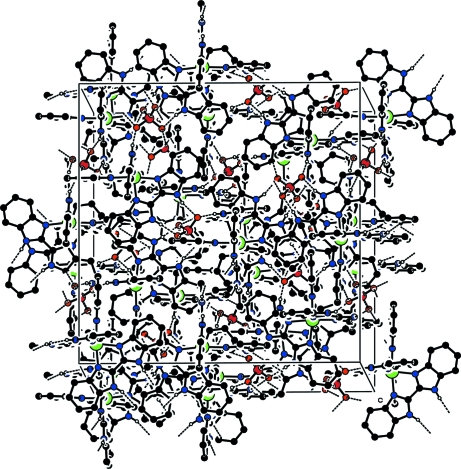
Three-dimensional structure of (I). Displacement ellipsoids are drawn at the 50% probability level. For clarity, H atoms not involved in hydrogen bonds are omitted.

## supplementary crystallographic information

### Comment

More attentions have been paid to metal-organic coordination compounds (MOCCs) because of their potential applications in gas absorption and separation, catalysis, nonlinear optics, luminescence and magnetism (Kitagawa & Matsuda 2007, Maspoch *et al.* 2007). In the field of coordination chemistry, the N,*N*-bidentate ligands, such as 2,2'-bipyridine, 1,10-phenanthroline and their derivatives act as versatile ligands owing to the stable coordination configuration in the bidentate N-donors chelating manner. Herein, we report the title compound (I).

The title compound (I) consists of four [Ni(C_14_H_10_N_4_)_3_]^2+^ complex cations, one [SO_4_]^2-^ and two [PO_4_]^3-^ anions. In the mlecular structure, the Ni^2+^ centre is coordinated by six N atoms from three bidentate 1H,1'*H*-2,2'-bi-1*H*-benzimidazole molecules (Fig.1). The 1H,1'*H*-2,2'-bi-1*H*-benzimidazole ligand was prepared *in situ* and coordinated to the Ni^2+^ cations in hydrothermal reaction. Additionally, the [SO_4_]^2-^ and [PO_4_]^3-^ anions statistically distribute in one position with 1/3 probability for S and 2/3 probability for P atoms. The environment of the Ni^2+^ caion is in a distorted octahedral geometry with the Ni—N distances ranging from 2.088 (3) to 2.122 (3) Å (Table 1).

In addition, the [Ni(C_14_H_10_N_4_)_3_]^2+^ complex cations, [SO_4_]^2-^ and [PO_4_]^3-^ anions in the complexes are linked together *via* many N—H···O hydrogen bonds resulting in a three-dimensional structural frameworks (Fig.2 and Table 2).

### Experimental

All reagents were commercially available and of analytical grade. The mixture of NiSO_4_.6H_2_O, H_3_PO_4_, oxalic acid, and 1,2-diaminobenzene in the mole ratio of 1: 1.5: 6: 6 was dissolved in 25 ml H_2_O, which was heated in a Teflon-lined steel autoclave inside a programmable electric furnace at 393 K for five days. After cooling the autoclave to room temperature, green block crystals of (I) were obtained.

### Refinement

H atoms were treated as riding, with C—H = 0.93 Å and N—H = 0.86 Å, and were refined as riding with *U*_iso_(H) = 1.2*U*_eq_(N and C).

### Crystal data

**Table:**

[Ni(C_14_H_10_N_4_)_3_]_4_(PO_4_)_2_(SO_4_)	*D*_x_ = 1.423 Mg m^−^^3^
*M**_r_* = 3331.96	Mo *K*α radiation, λ = 0.71073 Å
Cubic, *I*43*d*	Cell parameters from 3375 reflections
Hall symbol: I -4bd 2c 3	θ = 2.3–19.2°
*a* = 24.964 (7) Å	µ = 0.59 mm^−^^1^
*V* = 15558 (8) Å^3^	*T* = 296 K
*Z* = 4	Block, green
*F*(000) = 6872	0.32 × 0.27 × 0.23 mm

### Data collection

**Table:**

Bruker SMART CCD area-detector diffractometer	2551 independent reflections
Radiation source: fine-focus sealed tube	1782 reflections with *I* > 2σ(*I*)
graphite	*R*_int_ = 0.066
φ and ω scans	θ_max_ = 26.0°, θ_min_ = 2.0°
Absorption correction: multi-scan (SADABS; Sheldrick, 2001)	*h* = −30→11
*T*_min_ = 0.834, *T*_max_ = 0.876	*k* = −30→29
20222 measured reflections	*l* = −21→20

### Refinement

**Table:**

Refinement on *F*^2^	Secondary atom site location: difference Fourier map
Least-squares matrix: full	Hydrogen site location: inferred from neighbouring sites
*R*[*F*^2^ > 2σ(*F*^2^)] = 0.038	H-atom parameters constrained
*wR*(*F*^2^) = 0.098	*w* = 1/[σ^2^(*F*_o_^2^) + (0.0528*P*)^2^] where *P* = (*F*_o_^2^ + 2*F*_c_^2^)/3
*S* = 1.01	(Δ/σ)_max_ = 0.001
2551 reflections	Δρ_max_ = 0.61 e Å^−^^3^
177 parameters	Δρ_min_ = −0.20 e Å^−^^3^
0 restraints	Absolute structure: Flack (1983), 1182 Friedel pairs
Primary atom site location: structure-invariant direct methods	Flack parameter: −0.02 (2)

### Special details

**Table:**

Geometry. All e.s.d.'s (except the e.s.d. in the dihedral angle between two l.s. planes) are estimated using the full covariance matrix. The cell e.s.d.'s are taken into account individually in the estimation of e.s.d.'s in distances, angles and torsion angles; correlations between e.s.d.'s in cell parameters are only used when they are defined by crystal symmetry. An approximate (isotropic) treatment of cell e.s.d.'s is used for estimating e.s.d.'s involving l.s. planes.
Refinement. Refinement of *F*^2^ against ALL reflections. The weighted *R*-factor *wR* and goodness of fit *S* are based on *F*^2^, conventional *R*-factors *R* are based on *F*, with *F* set to zero for negative *F*^2^. The threshold expression of *F*^2^ > σ(*F*^2^) is used only for calculating *R*-factors(gt) *etc*. and is not relevant to the choice of reflections for refinement. *R*-factors based on *F*^2^ are statistically about twice as large as those based on *F*, and *R*- factors based on ALL data will be even larger.

### Fractional atomic coordinates and isotropic or equivalent isotropic displacement parameters (Å^2^)

**Table:**

	*x*	*y*	*z*	*U*_iso_*/*U*_eq_	Occ. (<1)
Ni1	0.564621 (16)	0.435379 (16)	−0.064621 (16)	0.0428 (2)	
P1	0.7500	0.6250	1.0000	0.0427 (4)	0.67
S1	0.7500	0.6250	1.0000	0.0427 (4)	0.33
N1	0.64773 (10)	0.44469 (11)	−0.06574 (11)	0.0443 (6)	
N2	0.71668 (10)	0.50117 (12)	−0.06248 (11)	0.0498 (7)	
H2B	0.7340	0.5309	−0.0604	0.060*	
N3	0.56297 (10)	0.42828 (11)	0.02009 (10)	0.0444 (6)	
N4	0.55652 (11)	0.37290 (12)	0.08942 (11)	0.0502 (7)	
H4B	0.5538	0.3435	0.1072	0.060*	
C1	0.69498 (13)	0.41559 (13)	−0.06917 (13)	0.0462 (8)	
C2	0.70405 (15)	0.36114 (16)	−0.07434 (15)	0.0594 (9)	
H2	0.6758	0.3370	−0.0763	0.071*	
C3	0.75637 (17)	0.34409 (18)	−0.07642 (16)	0.0744 (13)	
H3	0.7638	0.3077	−0.0797	0.089*	
C4	0.79858 (16)	0.3808 (2)	−0.07359 (16)	0.0707 (12)	
H4	0.8335	0.3679	−0.0750	0.085*	
C5	0.79104 (13)	0.43383 (18)	−0.06895 (15)	0.0627 (10)	
H5	0.8197	0.4576	−0.0670	0.075*	
C6	0.73810 (12)	0.45147 (14)	−0.06724 (14)	0.0473 (8)	
C7	0.66286 (13)	0.49523 (14)	−0.06164 (13)	0.0443 (8)	
C8	0.56226 (15)	0.45873 (13)	0.06673 (14)	0.0487 (9)	
C9	0.56566 (17)	0.51278 (15)	0.07424 (15)	0.0645 (11)	
H9	0.5681	0.5363	0.0455	0.077*	
C10	0.5653 (2)	0.53090 (18)	0.12671 (19)	0.0848 (13)	
H10	0.5672	0.5675	0.1333	0.102*	
C11	0.5623 (2)	0.49628 (19)	0.16920 (17)	0.0863 (14)	
H11	0.5627	0.5102	0.2037	0.104*	
C12	0.55859 (18)	0.44183 (18)	0.16272 (14)	0.0698 (11)	
H12	0.5562	0.4186	0.1917	0.084*	
C13	0.55865 (13)	0.42369 (14)	0.11042 (13)	0.0489 (8)	
C14	0.55946 (13)	0.37792 (14)	0.03571 (13)	0.0443 (8)	
O1	0.77314 (11)	0.59036 (9)	0.95604 (10)	0.0629 (7)	

### Atomic displacement parameters (Å^2^)

**Table:**

	*U*^11^	*U*^22^	*U*^33^	*U*^12^	*U*^13^	*U*^23^
Ni1	0.0428 (2)	0.0428 (2)	0.0428 (2)	−0.00140 (19)	−0.00140 (19)	0.00140 (19)
P1	0.0469 (6)	0.0341 (9)	0.0469 (6)	0.000	0.000	0.000
S1	0.0469 (6)	0.0341 (9)	0.0469 (6)	0.000	0.000	0.000
N1	0.0386 (14)	0.0485 (17)	0.0458 (16)	0.0000 (13)	−0.0006 (13)	0.0019 (15)
N2	0.0447 (17)	0.0543 (18)	0.0503 (17)	−0.0083 (14)	−0.0009 (14)	−0.0019 (15)
N3	0.0432 (16)	0.0445 (16)	0.0456 (15)	0.0006 (15)	−0.0046 (13)	−0.0007 (13)
N4	0.0552 (19)	0.0518 (18)	0.0436 (17)	−0.0046 (14)	−0.0034 (14)	0.0093 (14)
C1	0.049 (2)	0.0486 (19)	0.0411 (18)	0.0030 (16)	−0.0031 (17)	−0.0036 (15)
C2	0.054 (2)	0.060 (2)	0.065 (2)	0.0073 (18)	−0.0023 (19)	−0.0042 (19)
C3	0.064 (3)	0.076 (3)	0.084 (3)	0.024 (2)	−0.003 (2)	−0.009 (2)
C4	0.045 (2)	0.097 (4)	0.070 (3)	0.021 (2)	−0.002 (2)	−0.014 (2)
C5	0.0439 (19)	0.083 (3)	0.061 (3)	0.000 (2)	−0.0050 (18)	−0.016 (3)
C6	0.0417 (19)	0.064 (2)	0.0366 (18)	0.0018 (16)	−0.0048 (16)	−0.0077 (17)
C7	0.044 (2)	0.048 (2)	0.0410 (19)	−0.0065 (15)	−0.0027 (15)	−0.0012 (16)
C8	0.049 (2)	0.052 (2)	0.0449 (19)	0.0051 (16)	−0.0042 (18)	−0.0057 (16)
C9	0.092 (3)	0.050 (2)	0.051 (2)	−0.002 (2)	−0.009 (2)	−0.0036 (17)
C10	0.124 (4)	0.065 (3)	0.066 (3)	0.006 (3)	−0.010 (3)	−0.013 (2)
C11	0.133 (4)	0.077 (3)	0.049 (3)	0.001 (3)	−0.013 (3)	−0.017 (2)
C12	0.093 (3)	0.074 (3)	0.042 (2)	−0.006 (3)	−0.005 (2)	0.001 (2)
C13	0.050 (2)	0.055 (2)	0.0423 (19)	−0.0009 (18)	−0.0050 (16)	−0.0006 (16)
C14	0.0315 (18)	0.053 (2)	0.049 (2)	−0.0032 (16)	−0.0021 (15)	0.0045 (17)
O1	0.0818 (18)	0.0455 (15)	0.0616 (17)	−0.0077 (13)	0.0150 (14)	−0.0033 (12)

### Geometric parameters (Å, °)

**Table:**

Ni1—N1	2.088 (3)	C1—C6	1.401 (5)
Ni1—N1^i^	2.088 (3)	C2—C3	1.375 (5)
Ni1—N1^ii^	2.088 (3)	C2—H2	0.9300
Ni1—N3^ii^	2.122 (3)	C3—C4	1.398 (6)
Ni1—N3	2.122 (3)	C3—H3	0.9300
Ni1—N3^i^	2.122 (3)	C4—C5	1.342 (6)
P1—O1^iii^	1.512 (3)	C4—H4	0.9300
P1—O1^iv^	1.512 (3)	C5—C6	1.394 (4)
P1—O1	1.512 (3)	C5—H5	0.9300
P1—O1^v^	1.512 (3)	C7—C14^ii^	1.435 (5)
N1—C7	1.321 (4)	C8—C9	1.365 (5)
N1—C1	1.388 (4)	C8—C13	1.401 (5)
N2—C7	1.352 (4)	C9—C10	1.386 (6)
N2—C6	1.356 (4)	C9—H9	0.9300
N2—H2B	0.8600	C10—C11	1.370 (6)
N3—C14	1.319 (4)	C10—H10	0.9300
N3—C8	1.391 (4)	C11—C12	1.372 (6)
N4—C14	1.349 (4)	C11—H11	0.9300
N4—C13	1.373 (4)	C12—C13	1.382 (5)
N4—H4B	0.8600	C12—H12	0.9300
C1—C2	1.384 (5)	C14—C7^i^	1.435 (5)
			
N1—Ni1—N1^i^	95.67 (10)	C1—C2—H2	121.2
N1—Ni1—N1^ii^	95.67 (10)	C2—C3—C4	120.7 (4)
N1^i^—Ni1—N1^ii^	95.67 (10)	C2—C3—H3	119.6
N1—Ni1—N3^ii^	78.84 (10)	C4—C3—H3	119.6
N1^i^—Ni1—N3^ii^	170.67 (9)	C5—C4—C3	123.0 (4)
N1^ii^—Ni1—N3^ii^	92.40 (9)	C5—C4—H4	118.5
N1—Ni1—N3	92.40 (9)	C3—C4—H4	118.5
N1^i^—Ni1—N3	78.84 (10)	C4—C5—C6	116.6 (4)
N1^ii^—Ni1—N3	170.67 (9)	C4—C5—H5	121.7
N3^ii^—Ni1—N3	93.75 (9)	C6—C5—H5	121.7
N1—Ni1—N3^i^	170.67 (9)	N2—C6—C5	131.7 (3)
N1^i^—Ni1—N3^i^	92.40 (9)	N2—C6—C1	106.6 (3)
N1^ii^—Ni1—N3^i^	78.84 (10)	C5—C6—C1	121.7 (3)
N3^ii^—Ni1—N3^i^	93.75 (9)	N1—C7—N2	112.8 (3)
N3—Ni1—N3^i^	93.75 (9)	N1—C7—C14^ii^	118.2 (3)
O1^iii^—P1—O1^iv^	110.22 (17)	N2—C7—C14^ii^	128.9 (3)
O1^iii^—P1—O1	109.10 (9)	C9—C8—N3	130.9 (3)
O1^iv^—P1—O1	109.10 (9)	C9—C8—C13	121.0 (3)
O1^iii^—P1—O1^v^	109.10 (9)	N3—C8—C13	108.1 (3)
O1^iv^—P1—O1^v^	109.10 (9)	C8—C9—C10	116.9 (4)
O1—P1—O1^v^	110.22 (17)	C8—C9—H9	121.6
C7—N1—C1	105.2 (3)	C10—C9—H9	121.6
C7—N1—Ni1	112.9 (2)	C11—C10—C9	121.7 (4)
C1—N1—Ni1	141.9 (2)	C11—C10—H10	119.1
C7—N2—C6	107.0 (3)	C9—C10—H10	119.1
C7—N2—H2B	126.5	C10—C11—C12	122.5 (4)
C6—N2—H2B	126.5	C10—C11—H11	118.7
C14—N3—C8	105.8 (3)	C12—C11—H11	118.7
C14—N3—Ni1	112.0 (2)	C11—C12—C13	115.8 (4)
C8—N3—Ni1	142.1 (2)	C11—C12—H12	122.1
C14—N4—C13	107.0 (3)	C13—C12—H12	122.1
C14—N4—H4B	126.5	N4—C13—C12	131.5 (3)
C13—N4—H4B	126.5	N4—C13—C8	106.4 (3)
C2—C1—N1	131.2 (3)	C12—C13—C8	122.1 (3)
C2—C1—C6	120.4 (3)	N3—C14—N4	112.7 (3)
N1—C1—C6	108.4 (3)	N3—C14—C7^i^	117.7 (3)
C3—C2—C1	117.6 (4)	N4—C14—C7^i^	129.5 (3)
C3—C2—H2	121.2		
			
N1^i^—Ni1—N1—C7	−168.5 (2)	C2—C1—C6—N2	179.6 (3)
N1^ii^—Ni1—N1—C7	95.2 (3)	N1—C1—C6—N2	0.3 (4)
N3^ii^—Ni1—N1—C7	3.9 (2)	C2—C1—C6—C5	−1.8 (5)
N3—Ni1—N1—C7	−89.5 (3)	N1—C1—C6—C5	178.9 (3)
N3^i^—Ni1—N1—C7	41.7 (7)	C1—N1—C7—N2	0.4 (4)
N1^i^—Ni1—N1—C1	11.1 (4)	Ni1—N1—C7—N2	−179.9 (2)
N1^ii^—Ni1—N1—C1	−85.2 (3)	C1—N1—C7—C14^ii^	177.6 (3)
N3^ii^—Ni1—N1—C1	−176.5 (4)	Ni1—N1—C7—C14^ii^	−2.6 (4)
N3—Ni1—N1—C1	90.1 (4)	C6—N2—C7—N1	−0.2 (4)
N3^i^—Ni1—N1—C1	−138.6 (6)	C6—N2—C7—C14^ii^	−177.1 (3)
N1—Ni1—N3—C14	−100.0 (2)	C14—N3—C8—C9	178.8 (4)
N1^i^—Ni1—N3—C14	−4.7 (2)	Ni1—N3—C8—C9	−4.5 (7)
N1^ii^—Ni1—N3—C14	49.9 (7)	C14—N3—C8—C13	0.5 (4)
N3^ii^—Ni1—N3—C14	−178.9 (2)	Ni1—N3—C8—C13	177.2 (3)
N3^i^—Ni1—N3—C14	87.1 (3)	N3—C8—C9—C10	−178.2 (4)
N1—Ni1—N3—C8	83.5 (4)	C13—C8—C9—C10	0.0 (6)
N1^i^—Ni1—N3—C8	178.8 (4)	C8—C9—C10—C11	0.7 (7)
N1^ii^—Ni1—N3—C8	−126.7 (6)	C9—C10—C11—C12	−1.0 (8)
N3^ii^—Ni1—N3—C8	4.5 (4)	C10—C11—C12—C13	0.6 (8)
N3^i^—Ni1—N3—C8	−89.5 (3)	C14—N4—C13—C12	−178.1 (4)
C7—N1—C1—C2	−179.6 (4)	C14—N4—C13—C8	0.8 (4)
Ni1—N1—C1—C2	0.7 (7)	C11—C12—C13—N4	178.7 (4)
C7—N1—C1—C6	−0.4 (4)	C11—C12—C13—C8	0.0 (6)
Ni1—N1—C1—C6	180.0 (3)	C9—C8—C13—N4	−179.3 (3)
N1—C1—C2—C3	−179.6 (3)	N3—C8—C13—N4	−0.8 (4)
C6—C1—C2—C3	1.2 (5)	C9—C8—C13—C12	−0.3 (6)
C1—C2—C3—C4	−0.3 (6)	N3—C8—C13—C12	178.2 (4)
C2—C3—C4—C5	−0.2 (6)	C8—N3—C14—N4	0.0 (4)
C3—C4—C5—C6	−0.3 (6)	Ni1—N3—C14—N4	−177.9 (2)
C7—N2—C6—C5	−178.5 (4)	C8—N3—C14—C7^i^	−177.4 (3)
C7—N2—C6—C1	0.0 (4)	Ni1—N3—C14—C7^i^	4.8 (3)
C4—C5—C6—N2	179.5 (4)	C13—N4—C14—N3	−0.5 (4)
C4—C5—C6—C1	1.3 (6)	C13—N4—C14—C7^i^	176.5 (3)

Symmetry codes: (i) −*z*+1/2, −*x*+1, *y*−1/2; (ii) −*y*+1, *z*+1/2, −*x*+1/2; (iii) −*z*+7/4, −*y*+5/4, *x*+1/4; (iv) *z*−1/4, −*y*+5/4, −*x*+7/4; (v) −*x*+3/2, *y*, −*z*+2.

### Hydrogen-bond geometry (Å, °)

**Table:**

*D*—H···*A*	*D*—H	H···*A*	*D*···*A*	*D*—H···*A*
N4—H4B···O1^vi^	0.86	1.96	2.766 (4)	156
N2—H2B···O1^vii^	0.86	1.82	2.675 (4)	170

Symmetry codes: (vi) *x*−1/4, −*z*+5/4, −*y*+3/4; (vii) *x*, *y*, *z*−1.
